# Impact on carbon emissions of online study for a cohort of overseas students: A retrospective cohort study

**DOI:** 10.12688/f1000research.55156.5

**Published:** 2022-02-14

**Authors:** Richard F Heller, Ya-Yen Sun, Zhe Guo, Arunima Malik

**Affiliations:** 1 University of Newcastle, Newastle, NSW, Australia; 2 UQ Business School, The University of Queensland, Brisbane, Queensland, Australia; 3 The University of Sydney, Sydney, NSW, Australia; 4 ISA, School of Physics and Accounting, Business School, University of Sydney, Sydney, NSW, Australia

**Keywords:** Online learning, carbon emissions, airline travel, overseas students

## Abstract

**Background: **One of the benefits of online education is the potential reduction in carbon emissions through the decrease in travel to attend a university in person. We estimated the savings in CO _2 _emissions of an international cohort of master’s students who studied fully online from their home countries, rather than travelling to the UK and living there while attending university.

**Methods: **The city and country of residence of a cohort of students who first enrolled in the fully online Peoples-uni/Manchester Metropolitan University Master of Public Health programme between the second semester of 2011 and the first semester 2013 were recorded. Total difference in emissions was calculated by adding the estimated aviation emissions between Manchester, UK and the cities where students resided, to the difference in per capita emissions between the country of origin and the UK for the time that the student would have spent in Manchester, based on the semester in which they first enrolled.

**Results: **128 students enrolled from 70 cities in 30 countries. 93 students were from a range of African countries and 18 from the Indian sub-continent. Flights to and from Manchester were estimated to have accounted for 114,553kg of CO _2_ and living in Manchester for the duration of their course compared with staying in the home country would have been equivalent to 854,904kg of CO _2_. The combined net savings was 969,457kg of CO _2_.

**Conclusions: **A small cohort of overseas students, largely from Africa and India, studied online rather than attending university in the UK. The likely saving by this small cohort of nearly a million kg of CO _2 _emissions offers an indication of the potential environmental benefits of offering university education online to overseas students.

## Background

One of the benefits of online education is the potential reduction in carbon emissions through decreasing travel to attend university in person. Caird *et al*. ^1^ calculated that among 15 higher education institutions in the UK, distance-based education models achieved an 83% reduction in carbon emissions, with the fully online model achieving the lowest carbon emissions. Estimates included travel, purchase and use of ICT devices, purchase of books and publications and use of paper for printing, residential and home energy use and campus site operations. Versteijlen *et al*. conclude: “The introduction of online education allows [...] a huge reduction in carbon emissions and could thus help HEIs [Higher Education Institutions] to achieve their energy efficiency and sustainability goals” ^2^. However, there is little evidence in the existing literature that quantifies this potential for overseas students although one study reports that travel by overseas students to the university accounted for 6% of total emissions ^3^ and another, while describing a large variation in estimated carbon emissions between different universities in Texas, also estimated the carbon emissions from travel through a ‘study abroad’ programme in one university ^4^.

Peoples-uni, a volunteer led educational charity, provided fully online master’s level courses to health professionals in low- to middle-income countries (LMICs) from 2008 to 2021 ^5^. For four semesters between 2011 and 2013, a partnership allowed students to enrol in the Master of Public Health (MPH) offered by Manchester Metropolitan University (MMU) by solely online study through the Peoples-uni without travel to the UK.

This paper estimates the savings in CO _2_ emissions by this cohort of students who studied fully online from their home countries rather than travelling to and living in Manchester to attend the University in-person.

## Methods

A retrospective cohort study explored the records in the Peoples-uni database of each of the students who first enrolled through Peoples-uni in the MMU MPH award programme between the second semester of 2011 and the first semester of 2013. The city and country of residence were recorded, as was the final award gained. Even though the course was part-time, we assume that students would have been living in Manchester full-time and would have travelled by air from their home city. We assumed that they would have lived in Manchester for 18 months to complete a full 180 credit MPH, 12 months for those exiting with a 120 credit Graduate Diploma (passing all coursework except for the Dissertation) or 6 months for those exiting with a 60 credit Graduate Certificate (passing half the number of modules required for the Graduate Diploma). For students who passed some modules, but not enough to earn a Graduate Certificate, we assumed they would have spent 3 months in Manchester, and for those who passed no modules we assumed they would have withdrawn before travelling to Manchester. The dataset for this report can be found here ^6^.

The differences of carbon emissions during participation in the MMU MPH programme are calculated as the following:

Net emissions = (emissions of living in Manchester) – (emissions of living at home country) + round trip air transport emissions

If net emissions are larger than zero, this implies the online MMU MPH programme creates an environmental benefit - with a carbon footprint at home smaller than the footprint when living in Manchester combined with the air travel.

To calculate the difference, we first used the International Civil Aviation Organization (ICAO) carbon emissions calculator ^7^. The ICAO provides the comprehensive city-pair carbon dioxide emissions from air travel by taking into account aircraft types, route specific data, passenger load factors and cargo carried. We estimated the aviation emissions between Manchester, UK and the city where students resided. To avoid overestimating the environmental impact of the travel, we took a conservative approach by choosing the route with fewest number of stops and lowest flight time or miles where this was an option, even though these may not have been the cheapest options, nor the actual flights used by the students. Road travel from a city without an international airport was recorded but not included in a calculation of emissions as the mode of travel was unknown and the estimates would have been imprecise.

To calculate the emissions attributable to students living in the UK instead of their home country, the annual per capita CO _2_ emissions for each country were taken for the relevant years from data collected by the Carbon Dioxide Information Analysis Center and reported in OurWorldInData.org ^8^. The per capita emissions for the country of origin were subtracted from the per capita emissions for the UK over the time that the student would have spent in Manchester as a full-time student, starting with the semester in which they first enrolled. We did not estimate the carbon emissions associated with different educational processes themselves.

## Ethics statement

As part of the application process for entry to Peoples-uni courses, students were informed that their anonymised information might be used for research into the outcomes of the education programme. Data from the Peoples-uni database were extracted by one of the researchers (RFH) and de-identified by deleting the names of the students from the resulting spreadsheet shared for analysis with the other authors, and for the resulting publication. No ethical approval was sought due to the low-risk nature of the study.

## Results

From 2011 to 2013, 128 students enrolled in the MMU MPH programme from 70 cities in 30 countries, and travelled to Manchester by air from 55 cities. 93 students were from Africa and 18 from the Indian sub-continent.

94 students gained an MPH, from which we recorded an assumed 18 months living in Manchester, 9 gained a Graduate Diploma, equating to 12 months in Manchester, and 16 students gained a Graduate Certificate, equating to 6 months in Manchester. 5 students passed two modules, corresponding to 3 months in Manchester, and 4 students gained no passes and are assumed not to have travelled to Manchester at all.

34 students started in the second semester of 2011, 24 and 23 respectively in the first and second semesters 2012, and 47 in the first semester of 2013. Although all students were from LMICs, some were living in high-income countries at the start of their studies.

### Transport emissions

**Figure 1.**
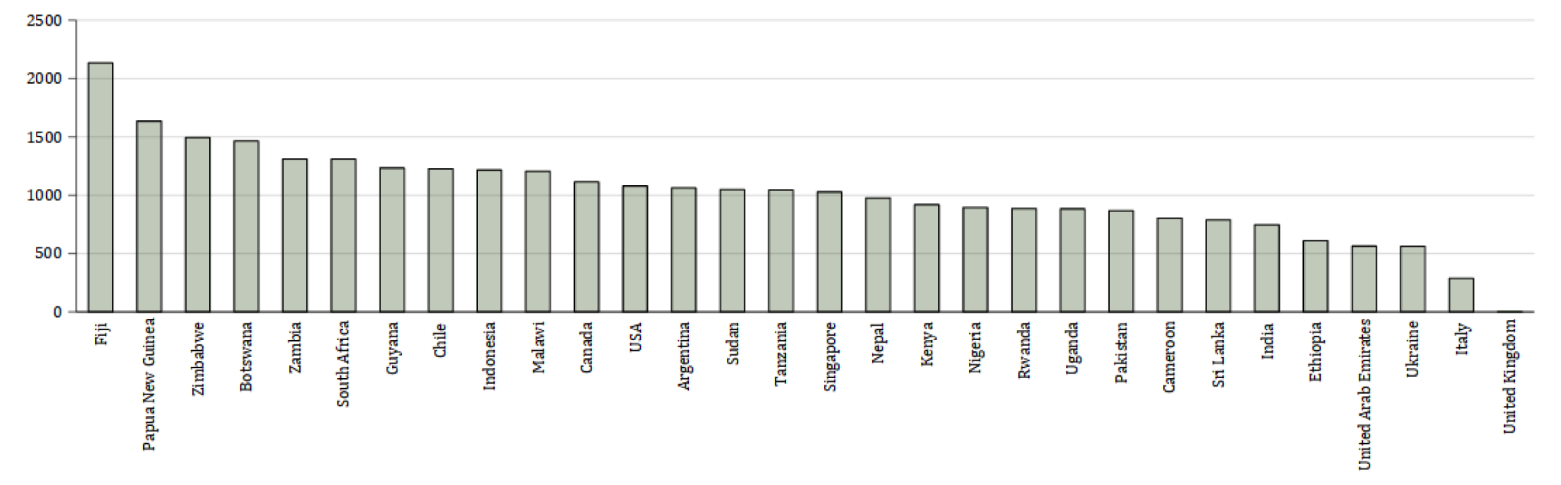
Mean emissions (in kg CO _2_) per country from air travel to Manchester and return. X axis shows the country from which the students come – where students come from more than one city in the country, the mean has been calculated to characterise the country. Y axis shows kg CO _2_.

Two students started the MPH programme in the UK, so were not counted in the calculation of transport emissions. Flights to and from Manchester were estimated to have accounted for 114, 553 kg of CO _2_ emissions, with an average of 924 kg per student. Transport emissions are largely determined by distance, and the largest emissions on flights were those flying intercontinental from Fiji (2,133 kg), Papua New Guinea (1,635 kg) and Zimbabwe (1,495 kg) to Manchester. Figure 1 shows the emissions for each country – where students came from more than one city in a country these were averaged to show country data.

### Emissions from living in Manchester

The two students who enrolled from the UK had no change in emissions, and eight students came from countries (South Africa, USA, Canada and United Arab Emirates) with higher emissions than in the UK, so contributed negative counts. Overall, the emissions per capita are linked strongly to national economic development status – the higher the wealth the larger the emission footprint. Because the MMU MPH programme was mainly offered to students from LMICs, students’ carbon footprint in their home country is generally lower than it would be living in Manchester, although this will vary over time. As examples, the net per capita CO _2_ emission estimates used for 2013 were 7,354 kg for Manchester, 103 kg for Ethiopia and 72 for Rwanda. For the group as a whole, living in Manchester for the duration of their course compared with staying in the home country would have been equivalent to a net excess of 854,904 kg of CO _2_.

**Figure 2.**
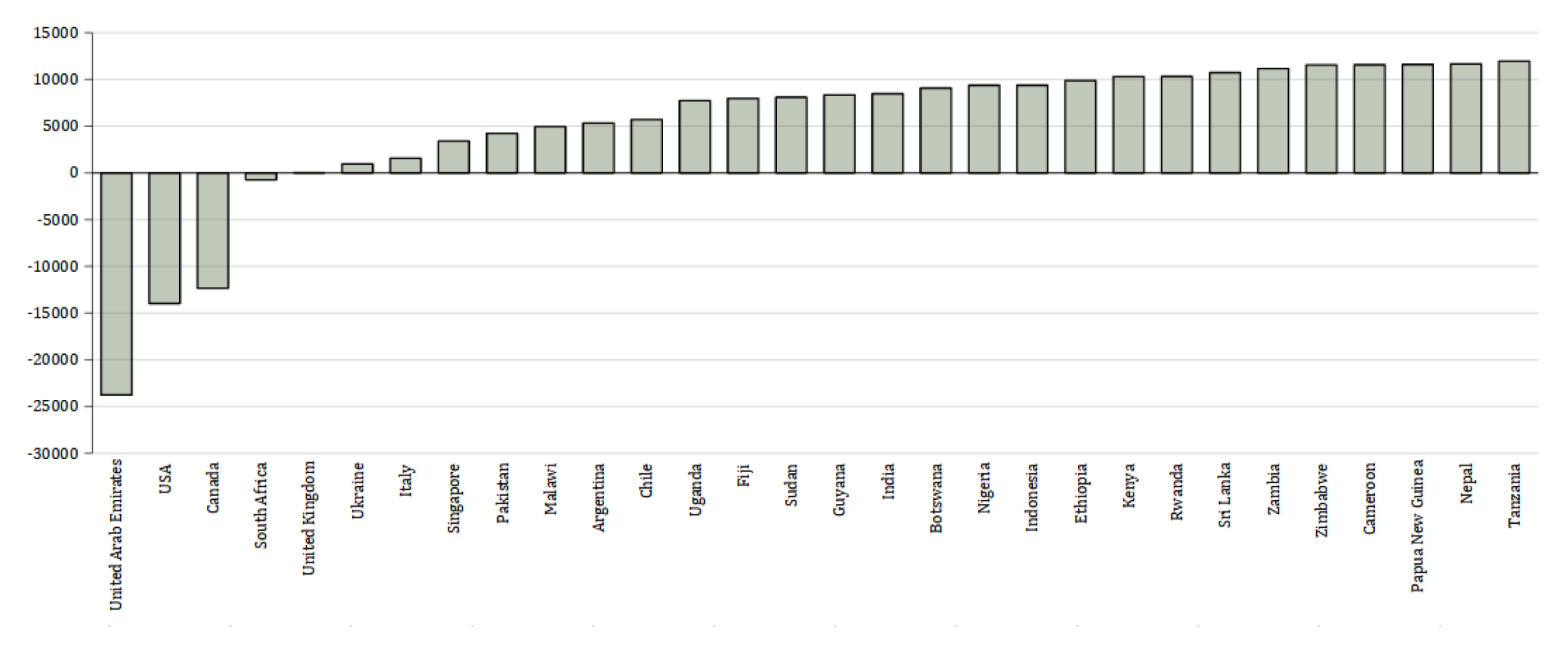
Difference in net emissions (kg CO _2_) between physical attendance in Manchester and studying online at home. X axis shows the country from which the students come. Y axis shows the net difference in emissions between the UK and each country in kg CO _2_.

Combining transport and living gives an estimate of total excess net emissions of 969,457 kg of CO _2_. Figure 2 shows the total net emissions per country.

## Discussion

This cohort of 128 master’s students was estimated to have saved 969,457 kg of CO _2_ through studying online from their home country rather than travelling to and living in Manchester, UK to attend in person.

We used conservative assumptions for flight estimations in terms of number of stops and routes taken, and also assumed that the students travelled alone without family and did not return home during the programme. Flight emissions may reduce over time with increasing global attention to the climate change issue, due to technological increases in aircraft efficiency or aircraft emission standards. However it should be noted that such changes have not yet made any notable difference to total global emissions, probably due to the 'Jevons Paradox' as demand increases. Per capita emissions will also change over time in different ways across countries.

We have assumed that a student living in Manchester would have the same consumption patterns as the general population, and so created our method of calculating their consumption by subtracting the per capita CO _2_ emissions of their own country from that of the UK. It may be that students have lower consumption patterns than the general population, although the university campus has a high carbon footprint ^9, 10^. Caird *et al*. ^1^ estimate 36kg CO _2_ per 100 study hours for UK based fully online courses (compared with 278kg for face-to-face teaching). Applying this estimate for online teaching to our cohort would equate to 648kg over the course of the master’s degree, and 68,796kg for the whole cohort. However, it is difficult to apply this to Peoples-uni which did not have a campus, used Open Educational Resources and whose students live in LMICs. Future research could consider specific supply chain aspects for quantifying reductions in emissions from online learning relating to students from LMICs. To this end, a global multi-regional input-output model could be integrated with detailed information on expenditure patterns of students on a country-by-country basis for quantifying their at-home carbon footprint, and the footprint if they travelled to the UK. Such an analysis could be performed at a sector-level, enabling the quantification of hotspots. Future work could also focus on expanding such an assessment to university-wide quantification of emission savings from online learning, beyond the assessed master’s programme in this work, to provide an accurate estimate of the emissions from different teaching models related to the ‘export’ of higher education to LMIC populations. A university-wide assessment could also include savings through online working of the teaching staff, a possible decrease in electricity consumption in lecture theatres (and possible increase from students’ perspective). Future research could also consider the costs associated with the sourcing of equipment for accessing online material, such as laptops, internet plans, and associated carbon emissions.

## Conclusion

Project Atlas, quoting UNESCO data, estimated in their 2020 report that there were more than 5.6 million higher education students globally that were studying abroad ^11^. In each of the top three countries receiving overseas students, the United States, the United Kingdom and Canada, more than 20% of all students were international. In the UK in 2019/20 there were more than 250,000 postgraduate non-UK students, the majority from outside the EU ^12^. Considering that the countries from which most overseas students come have lower emissions per capita, having international students enrolled in in-person programmes will create a net emission increase compared with online-study. Given the large number of overseas students globally, their impact on carbon emissions is considerable.

There is a literature on the way in which numerous international economic activities affect the environment ^13, 14^ and the importance of international education to the economy of many countries demonstrates the value of considering how online education might contribute to a reduction in global CO _2_ emissions.

The benefits of reducing CO _2_ emissions through online education for international students should be seen in the context of the COVID era, which has demonstrated the importance of online education and the limits to international travel. Despite the very important emissions benefit of online learning, there is likely to be an academic, social, and cultural benefit to international travel for these MPH students which has been forgone due to COVID-19. However, this should be seen in the context of the increased global availability of education delivered online without the need for, and costs of, international travel, and demonstrates the importance of the systems within which we live that determine the per capita carbon emissions that are attributable to us. That even a small cohort of international students, largely from Africa and India, studying online rather than travelling to the UK likely saved nearly a million kg of CO _2_ provides an indication of the extent of the savings that could be made through the development of online education for overseas students.

## Data availability

### Underlying data

Data come from International Civil Aviation Organization (ICAO) carbon emissions calculator ^7^ https://www.icao.int/environmental-protection/CarbonOffset/Pages/default.aspx; and CO _2_ and Greenhouse Gas Emissions ^8^ https://ourworldindata.org/co2-and-other-greenhouse-gas-emissions

Zenodo: Saving carbon emissions through online learning for overseas students [Data set] https://doi.org/10.5281/zenodo.5335866 ^6^

This project contains the following underlying data:

- Student data and calculations, and CO _2_ emissions data

Data are available under the terms of the Creative Commons Attribution 4.0 International license (CC-BY 4.0).
