# Supplementary material for: Impact on carbon emissions of online study for a cohort of overseas students: A retrospective cohort study
Source: F1000Res. 2022 Feb 14;10:849. Originally published 2021 Aug 25. [Version 5] doi: 10.12688/f1000research.55156.5 (PMC8764561; doi:10.12688/f1000research.55156.5)
Supplement: Peer review history

# Peer review: Reviewer response for version 3

**DOI:** 10.5256/f1000research.77856.r119728

**Published:** 2022-01-17

**Authors:** Geoffrey R Browne

**Affiliations:** 1 Melbourne School of Design, University of Melbourne, Melbourne, Vic, Australia

This is a concise paper about a neat study that estimates, under conditions of limited data and therefore some reasonable assumptions, the emission reductions attributable to online as opposed to face to face learning, made up of 1) flights and 2) cost of living emissions attributable to a country's systems. Generally, online learning resulted in a reduction in emissions attributable to the cohort, with some variety in this depending on students' home country.

 The limitations are explicit, which for such a study is important, and the findings have verisimilitude. Most of my comments take a broader picture which if the authors decide to use, could round out the article nicely,  For example:

- Despite the very important emissions benefit of online learning, there is likely to be an academic, social, and cultural benefit to international travel for these MPH students which has been forgone due to COVID-19. My own experience teaching in an online MPH (and other masters) is that for some students, there is lower engagement and possibly poorer academic performance. If the authors have experience or evidence of this that they could mention briefly, I feel it would round out the paper nicely.

ABSTRACT

- 'MPH' should be expanded in the first instance.
- Methods: This part is currently oddly written and unclear about what you did. Important to get this right in the Abstract. It reads as though you estimated emissions attributable to flights, but then ignored these and then subtracted per capita emissions attributable to living in the UK while (Cf. 'over the time'?) studying from per capita emissions of the country of origin. This is incorrect so you need to clarify this. How about this? "Total difference in emissions was calculated by adding the estimated aviation emissions between Manchester, UK and the cities where students resided, to the difference in per capita emissions (generally a reduction) between the country of origin and the UK for the time that the student would have spent in Manchester, based on the semester in which they first enrolled.".
- Also, the authors might find the word 'attributable' useful when redrafting, it can be a more efficient way of communicating exactly what you are including in your estimates. For example, "To calculate the emissions of students living in the UK compared with their home country..." can be improved by writing, "To calculate the emissions attributable to students living in the UK instead of their home country...".

METHODS; data sources

- No inferential statistical analyses were performed.
- Overall, methods seem sound. I am not an expert in the details of carbon accounting, specifically, whether databases like International Civil Aviation Organization (ICAO) carbon emissions calculator and OurWorldInData.org (is this the 'original' source?) are the best sources. They seem reputable but I will leave this to others to review.

RESULTS

- Re: "Overall, the emissions per capita are linked strongly to national economic development status – the higher the wealth the larger the emission footprint." It is great that the authors make this explicit. I'd like to see a bit more on this; a statement that makes it clear that the systems within which live significantly determine the p/c emissions that are attributable to us, and so it is systems change, rather than individual behaviour change, that is needed, and that systems change is an issue of international ethical responsibility for people in power, rather than preventing or limiting international students from benefiting from the in-person educational opportunities provided in the global north (not that you are recommending this, but it could be read that way). This might be a good point for the conclusion.

DISCUSSION

- Re: "flight emissions may reduce over time with increasing global attention to the climate change issue". I suggest you say very briefly (like, a few words only) what the mechanism might be: technological increases in aircraft efficiency? Aircraft emission standards? Also, please note that such tech and policy changes have not yet made any notable difference to total global emissions, probably due to the 'Jevons Paradox'.

Is the work clearly and accurately presented and does it cite the current literature?

Partly

If applicable, is the statistical analysis and its interpretation appropriate?

Not applicable

Are all the source data underlying the results available to ensure full reproducibility?

Yes

Is the study design appropriate and is the work technically sound?

Yes

Are the conclusions drawn adequately supported by the results?

Yes

Are sufficient details of methods and analysis provided to allow replication by others?

Partly

Reviewer Expertise:

Sustainability, urban planning, public health, pedagogy

I confirm that I have read this submission and believe that I have an appropriate level of expertise to confirm that it is of an acceptable scientific standard.

# Response

**Published:** 2022-01-18

**Authors:** Richard F Heller

**Affiliations:** University of Newcastle, Australia, Australia

Thank you for this review. We have submitted a revised version of the paper incorporating each of your suggestions.

# Peer review: Reviewer response for version 3

**DOI:** 10.5256/f1000research.77856.r95409

**Published:** 2021-10-04

**Authors:** Andy Lane, Sally Caird

**Affiliations:** 1 The Open University, Milton Keynes, UK; 2 School of Engineering and Innovation, Faculty of Science, Technology, Engineering and Maths, The Open University, Milton Keynes, UK

We do not feel inclined to change our assessment despite the changes made.

 The authors have responded to most of our criticisms but do not go as far as openly admitting it is purely about travel impacts that just happen to be applied to an HE case. Equally, I feel that the authors have misrepresented our critique when they say:

*“We have also added the following to the Discussion: “Caird et al. 1 estimate 36kg CO2 per 100 study hours for UK based fully online courses (compared with 278kg for face-to-face teaching). Applying this estimate for online teaching to our cohort would equate to 648kg over the course of the master’s degree, and 68,796kg for the whole cohort. However, it is difficult to apply this to Peoples-uni which did not have a campus, used Open Educational Resources and whose students* *live in LMICs.”*

 First, this does not acknowledge that our data only had a few international students in it so travel impacts were less than for their study, while this statement still persists in drawing a very tight boundary around what is included in the calculations. Peoples-uni might not have a campus but the people involved in running the course have residential impacts while the course was on the University of Manchester books and that has campus-based impacts, and producing OERs has impacts.

Is the work clearly and accurately presented and does it cite the current literature?

Partly

If applicable, is the statistical analysis and its interpretation appropriate?

Partly

Are all the source data underlying the results available to ensure full reproducibility?

Partly

Is the study design appropriate and is the work technically sound?

Partly

Are the conclusions drawn adequately supported by the results?

Partly

Are sufficient details of methods and analysis provided to allow replication by others?

Yes

Reviewer Expertise:

NA

We confirm that we have read this submission and believe that we have an appropriate level of expertise to confirm that it is of an acceptable scientific standard, however we have significant reservations, as outlined above.

# Response

**Published:** 2021-10-06

**Authors:** Richard F Heller

**Affiliations:** University of Newcastle, Australia, Australia

We are disappointed that the reviewers do not feel that our extensive revisions have met their initial reservations, as we agreed with the thrust of their comments and attempted to meet them responsibly. We are confused by the statement by the reviewers that we " *...do not go as far as openly admitting it is purely about travel impacts that just happen to be applied to an HE case*". In fact, as the reviewers note in their summary of our methods in their initial review, it is more than travel, as the students who travel to the UK have to live there, mostly for 18 months, during which time their carbon emissions are higher than they would have been if they had stayed in their home countries. This is related to their living, transport, and consumption patterns. We said " *We have assumed that a student living in Manchester would have the same consumption patterns as the general population, and so created our method of calculating their consumption by subtracting the per capita CO _2_ emissions of their own country from that of the UK. It may be that students have lower consumption patterns than the general population, although the university campus has a high carbon footprint ^9, 10^* ". We believe that we have been very clear in adding to the Methods section that *“We did not estimate the* *carbon emissions associated with different* *educational processes themselves.*” and the first sentence of the Discussion states: “ *This cohort of 128 master’s students was estimated to have saved 969,457 kg of CO _2_ through studying online from their home country rather than travelling to and living in Manchester, UK to attend in person.”*

 In the Discussion, we have also extensively listed the requirements of a future more detailed study than in this brief report to take into account the additional specific emissions related to higher education: “ *Future work could also focus on expanding such an assessment to university-wide quantification of emission savings from online learning, beyond the assessed master’s programme in this work, to provide an* *accurate estimate of the emissions from different teaching models related to the* ‘ *export’ of higher education to LMIC populations.”.*

 We are surprised by the reviewers’ second suggestion that we " *have misrepresented our critique*". Our intention was to acknowledge the published work of the reviewers by a direct quote from their study to show that it is possible to calculate the detailed impact of the higher education environment on carbon emissions, but to say that this result could not be applied directly to our situation both due to the special nature of our educational process and to the fact that students came from LMICs. This was to link to the addition of details we had included in the Background about the Caird *et al.* study and set the scene for the future work as described above.

 We hope that the reviewers might reconsider their assessment of our revisions.

# Peer review: Reviewer response for version 3

**DOI:** 10.5256/f1000research.77856.r95408

**Published:** 2021-09-24

**Authors:** Vincent Tawiah

**Affiliations:** 1 DCU Business School, Dublin City University, Dublin, Ireland

The paper is okay for indexing.

Is the work clearly and accurately presented and does it cite the current literature?

Partly

If applicable, is the statistical analysis and its interpretation appropriate?

Not applicable

Are all the source data underlying the results available to ensure full reproducibility?

Yes

Is the study design appropriate and is the work technically sound?

Yes

Are the conclusions drawn adequately supported by the results?

Yes

Are sufficient details of methods and analysis provided to allow replication by others?

Yes

Reviewer Expertise:

Climate change

I confirm that I have read this submission and believe that I have an appropriate level of expertise to confirm that it is of an acceptable scientific standard.

# Peer review: Reviewer response for version 2

**DOI:** 10.5256/f1000research.77395.r93060

**Published:** 2021-09-15

**Authors:** Andy Lane, Sally Caird

**Affiliations:** 1 The Open University, Milton Keynes, UK; 2 School of Engineering and Innovation, Faculty of Science, Technology, Engineering and Maths, The Open University, Milton Keynes, UK

The introduction is quite poor in setting the context. Some material in the conclusions should be in the introduction, for example, the discussion of Caird S, Lane A, Swithenby E, *et al.*: Design of higher education teaching models and carbon impacts. Int J Sust Higher Ed. 2015; 16(1): 96–111 ^1^ would help with that context rather than being an afterthought in the conclusions.

 The method used in this study is very high level and crude. It ignores the HE context (in terms of HE systems, climate action and sustainability policies and GHG emissions data) and does not describe the two teaching models compared; so consequently, it offers no insights into systemic aspects of HE institutional systems and teaching models. In other words, the method would be no different if it were calculating the effects of visiting the UK on holidays or for work for the same duration, and so it offers limited insight from a HE perspective. The title is therefore misleading because it does not look at the impacts of ‘online and in-person study’, instead, it estimates the impact of air travel of overseas students.

 As far as we can tell, limited primary data was collected, other than on the number of students on a programme, their nationality, and their qualification programme. The method does not take student or staff activities related to teaching and learning into account, other than to make assumptions on average behaviours rather than any self-reporting of 'actual' behaviours. The study assumes:

- students living in Manchester would have the same consumption patterns as the general population in the UK (is there no variation in consumption within countries?);
- students would have been living in Manchester full-time;
- the length of time students would have lived in Manchester, based on the time needed to complete their qualification programmes;
- students would have travelled by air from their home city;
- flight impacts in terms of the number of stops and routes taken;
- students travelled alone without family and did not return home during the programme.

These limitations should be acknowledged, i.e. the study does not consider HE systems nor students specifically, and so the study is just an estimation of travel emissions related to visitors to the UK for a specific period of time.

Is the work clearly and accurately presented and does it cite the current literature?

Partly

If applicable, is the statistical analysis and its interpretation appropriate?

Partly

Are all the source data underlying the results available to ensure full reproducibility?

Partly

Is the study design appropriate and is the work technically sound?

Partly

Are the conclusions drawn adequately supported by the results?

Partly

Are sufficient details of methods and analysis provided to allow replication by others?

Yes

Reviewer Expertise:

Environmental systems, innovation

We confirm that we have read this submission and believe that we have an appropriate level of expertise to confirm that it is of an acceptable scientific standard, however we have significant reservations, as outlined above.

## References

[1] : Design of higher education teaching models and carbon impacts. *International Journal of Sustainability in Higher Education* .2015;16(1) : 10.1108/IJSHE-06-2013-0065 96-111 10.1108/IJSHE-06-2013-0065

# Response

**Published:** 2021-09-21

**Authors:** Richard F Heller

**Affiliations:** University of Newcastle, Australia, Australia

​Reviewer Report

 Andy Lane, The Open University, Milton Keynes, UK

 Sally Caird, The Open University, Milton Keynes, UK

 The introduction is quite poor in setting the context. Some material in the conclusions should be in the introduction, for example, the discussion of Caird S, Lane A, Swithenby E, *et al.*: Design of higher education teaching models and carbon impacts. Int J Sust Higher Ed. 2015; 16(1): 96–111 ^1^ would help with that context rather than being an afterthought in the conclusions.

***Author response: **We have quoted this paper at the start of the Background which now reads as follows:* *“One of the benefits of online education is the potential reduction in carbon emissions through decreasing travel to attend university in person. Caird et al.* ^*1*^ *calculated that among 15 higher education institutions in the UK, distance-based education models achieved an 83% reduction in carbon emissions, with the fully online model achieving the lowest carbon emissions. Estimates included travel, purchase and use of ICT devices, purchase of books and publications and use of paper for printing, residential and home energy use and campus site operations. Versteijlen et al. conclude: “The introduction of online education allows [...] a huge reduction in carbon emissions and could thus help HEIs [Higher Education Institutions] to achieve their energy efficiency and sustainability goals”* ^*2*^ *”*

 The method used in this study is very high level and crude. It ignores the HE context (in terms of HE systems, climate action and sustainability policies and GHG emissions data) and does not describe the two teaching models compared; so consequently, it offers no insights into systemic aspects of HE institutional systems and teaching models. In other words, the method would be no different if it were calculating the effects of visiting the UK on holidays or for work for the same duration, and so it offers limited insight from a HE perspective. The title is therefore misleading because it does not look at the impacts of ‘online and in-person study’, instead, it estimates the impact of air travel of overseas students.

***Author response: **To reflect this comment* *w* *e have changed the title as follows: “Impact on carbon emissions of online study for a cohort of overseas students: A retrospective cohort study”.*

*We have also added to the Methods the following: “* *We did not estimate* *the* *carbon emissions associated with* *different* *educational processes* *themselves* *.* *”*

*We have also added the following to the Discussion: “Caird et al.* ^*1*^ *estimate 36kg CO*_*2*_ *per 100 study hours for UK based fully online courses (compared with 278kg for face-to-face teaching). Applying this estimate for online teaching to our cohort would equate to 648kg over the course of the master’s degree, and 68,796kg for the whole cohort.* *However, it is difficult to apply this to Peoples-uni w* *hich did not have a* *campus* *,* *use* *d* *Open Educational Resources and whose students live in LMICs.* *”*

*We have also added to our original sentence in the Discussion “Future work could also focus on expanding such an assessment to university-wide quantification of emission savings from online learning, beyond the assessed master’s programme in this work.”* *which now reads* *: “Future work could also focus on expanding such an assessment to university-wide quantification of emission savings from online learning, beyond the assessed master’s programme in this work,* *to provide a* *n* *accurate estimate of the* *emissions from* *different* *teaching* *models* *related to the* *‘export’ of higher education to LMIC populations* *.”*

*We have also changed the final sentence in the Abstract from “The saving by this small cohort of nearly a million kg of CO *_*2*_ *emissions* *through not attending university in person* *offers an indication of the potential environmental benefits of offering university education online to overseas students.” to “The* *likely* *saving by this small cohort of nearly a million kg of CO *_*2*_ *emissions offers an indication of the potential environmental benefits of offering university education online to overseas students.”*

 As far as we can tell, limited primary data was collected, other than on the number of students on a programme, their nationality, and their qualification programme. The method does not take student or staff activities related to teaching and learning into account, other than to make assumptions on average behaviours rather than any self-reporting of 'actual' behaviours. The study assumes:

- students living in Manchester would have the same consumption patterns as the general population in the UK (is there no variation in consumption within countries?);
- students would have been living in Manchester full-time;
- the length of time students would have lived in Manchester, based on the time needed to complete their qualification programmes;
- students would have travelled by air from their home city;
- flight impacts in terms of the number of stops and routes taken;
- students travelled alone without family and did not return home during the programme.

These limitations should be acknowledged, i.e. the study does not consider HE systems nor students specifically, and so the study is just an estimation of travel emissions related to visitors to the UK for a specific period of time.

***Author response: **We thank the Reviewers for these suggestions which clarify the nature of the study. As indicated above, we have now acknowledged the limitations of the study at various points in the paper, including in the title,* *and feel that the changes more accurately reflect the nature of the study and* *any* *implications.*

# Peer review: Reviewer response for version 2

**DOI:** 10.5256/f1000research.77395.r93064

**Published:** 2021-09-14

**Authors:** Vincent Tawiah

**Affiliations:** 1 DCU Business School, Dublin City University, Dublin, Ireland

This paper addresses an interesting and relevant topic on how online education could solve environmental problems. I suggest the authors improve the paper by considering the following points:

- Given that online education covers international activity, I suggest that the authors provide more literature on how international activities affect the environment and then narrow it down to online education. The authors could consider the following papers:
  - The environmental footprint of China-Africa engagement by Tawiah *et al*. (2020) ^1^;
  - Energy resource melioration and CO2 emissions in China and Nigeria; Efficiency and trade perspective by Li *et al.* (2020) ^2^.
- Policy implication: COVID-19 has forced many HEI to move online; hence, I expect the authors to provide some policy implications on findings nested with the COVID-19 situation. One way is to articulate how online education could reduce emissions and make your studies more relevant in this COVID era.

All the best.

Is the work clearly and accurately presented and does it cite the current literature?

Partly

If applicable, is the statistical analysis and its interpretation appropriate?

Not applicable

Are all the source data underlying the results available to ensure full reproducibility?

Yes

Is the study design appropriate and is the work technically sound?

Yes

Are the conclusions drawn adequately supported by the results?

Yes

Are sufficient details of methods and analysis provided to allow replication by others?

Yes

Reviewer Expertise:

Environment

I confirm that I have read this submission and believe that I have an appropriate level of expertise to confirm that it is of an acceptable scientific standard, however I have significant reservations, as outlined above.

## References

[1] : The environmental footprint of China-Africa engagement: An analysis of the effect of China - Africa partnership on carbon emissions. *Sci Total Environ* .2021;756: 10.1016/j.scitotenv.2020.143603 143603 10.1016/j.scitotenv.2020.143603 33248792

[2] : Energy resource melioration and CO2 emissions in China and Nigeria: Efficiency and trade perspectives. *Resources Policy* .2020;68: 10.1016/j.resourpol.2020.101769 10.1016/j.resourpol.2020.101769

# Response

**Published:** 2021-09-21

**Authors:** Richard F Heller

**Affiliations:** University of Newcastle, Australia, Australia

​Reviewer Report

 Vincent Tawiah, DCU Business School, Dublin City University, Dublin, Ireland 

 This paper addresses an interesting and relevant topic on how online education could solve environmental problems. I suggest the authors improve the paper by considering the following points:

- Given that online education covers international activity, I suggest that the authors provide more literature on how international activities affect the environment and then narrow it down to online education. The authors could consider the following papers:
  - The environmental footprint of China-Africa engagement by Tawiah *et al*. (2020) ^1^ ;
  - Energy resource melioration and CO2 emissions in China and Nigeria; Efficiency and trade perspective by Li *et al.* (2020) ^2^ .

***Author response: **We have added both of these references, and added the following to the Conclusion: “There is a literature on the way in which numerous* *international* *economic* *activities affect the environment* ^*13,14*^ *and* *the importance of international education to the economy of many countries demonstrates the value of considering how online education might contribute to a reduction in global CO*_*2*_ *emissions.* *”*

- Policy implication: COVID-19 has forced many HEI to move online; hence, I expect the authors to provide some policy implications on findings nested with the COVID-19 situation. One way is to articulate how online education could reduce emissions and make your studies more relevant in this COVID era.

***Author response: **We have added the following to the Conclusion: “The benefits of reducing CO*_*2*_ *emissions through online* *education for international students should be seen in the context of the COVID era, which has demonstrated the importance of online education and the limits to international travel.* *”*

 All the best.

***Author response: **Many thanks for your helpful suggestions. We hope that the corrections we have made capture your points.*
